# Reliability and Discriminant Validity of a Checklist for Surgical Scrubbing, Gowning and Gloving

**DOI:** 10.5195/ijms.2021.1221

**Published:** 2022-04-05

**Authors:** Stephen P. Canton, Christine E. Foley, Isabel Fulcher, Laura K. Newcomb, Noah Rindos, Nicole M. Donnellan

**Affiliations:** 1 Department of Orthopaedic Surgery, University of Pittsburgh School of Medicine, UPMC, Pittsburgh, United States; 2 Department of Obstetrics and Gynecology, The Warren Alpert Medical School of Brown University, Providence, RI, United States; 3 Department of Global Health and Social Medicine, Harvard Medical School, Boston, MA, United States; 4 Department of Obstetrics and Gynecology, University of Virginia School of Medicine, Charlottesville, VA, United States; 5 Department of Obstetrics and Gynecology, Allegheny General Hospital, Pittsburgh, PA, United States; 6 Department of Obstetrics, Gynecology and Reproductive Sciences, UPMC Magee-Womens Hospital, Pittsburgh, PA, United States

**Keywords:** Medial Education, Surgery, Augmented Reality, Virtual Reality (Source: MeSH-NLM)

## Abstract

**Background:**

Surgical scrubbing, gowning, and gloving is challenging for medical trainees to learn in the operating room environment. Currently, there are few reliable or valid tools to evaluate a trainee’s ability to scrub, gown and glove. The objective of this study is to test the reliability and validity of a checklist that evaluates the technique of surgical scrubbing, gowning and gloving (SGG).

**Methods:**

This Institutional Review Board-approved study recruited medical students, residents, and fellows from an academic, tertiary care institution. Trainees were stratified based upon prior surgical experience as novices, intermediates, or experts. Participants were instructed to scrub, gown and glove in a staged operating room while being video-recorded. Two blinded raters scored the videos according to the SGG checklist. Reliability was assessed using the intraclass correlation coefficient for total scores and Cohen’s kappa for item completion. The internal consistency and discriminant validity of the SGG checklist were assessed using Cronbach alpha and the Wilcoxon rank sum test, respectively.

**Results:**

56 participants were recruited (18 novices, 19 intermediates, 19 experts). The intraclass correlation coefficient demonstrated excellent inter-rater reliability for the overall checklist (0.990), and the Cohen’s kappa ranged from 0.598 to 1.00. The checklist also had excellent internal consistency (Cronbach’s alpha 0.950). A significant difference in scores was observed between all groups (p < 0.001).

**Conclusion:**

This checklist demonstrates a high inter-rater reliability, discriminant validity, and internal consistency. It has the potential to enhance medical education curricula.

## Introduction

Surgical scrubbing, gowning and gloving (SGG) are fundamental skills required to safely participate in surgery. These skills are challenging for medical trainees to master due to the learning environment in the operating room (OR). The rapid pace, limited time, and unavailability of expert medical professionals to provide training, hierarchy and the pressure of the high-stakes clinical environment are contributing factors to the OR culture.^1-4^ Such factors obstruct trainee skill acquisition and increase trainee stress, which negatively impacts the learning environment in the OR.^1,4-6^ Simulation-based education is rapidly gaining momentum, aligning with the paradigm shift in medical education as it transitions from “see one, do one, teach one” to a deliberate practice model.^4-7^ A SSG simulation model can provide an opportunity to prepare students and mitigate stress while in the OR.

The first step in developing simulation or assessment tools is formulating the content of the training that underlies the instruction. Checklists are commonly used in medical education to evaluate clinical skills in a simulated environment.^7-11^ Checklists standardize procedural training, provide an objective assessment to track progression, and can be used as an assessment tool to determine competency or suggest remediation.^12^ Educational checklists have high inter-rater reliability and trainee discrimination which allows for quality feedback for the learner. Compared to global rating scales, checklists have also been shown to require less rater training.^13^

There are very few reliable or valid tools for evaluating a trainee’s ability to scrub, gown and glove,^14^ and the few published studies lack methodologic rigor justifying the development of procedural checklists.^3,15,16^ The objective of this study was to assess the reliability and validity of this SGG checklist by assessing inter-rater reliability, internal consistency, and construct (discriminant) validity. We hypothesize that this tool will be able to detect a difference in skills between learners with different levels of surgical experience.

## Methods

### Study Design and Participants

This is a cross-sectional study to assess the validity and repeatability of a checklist created to evaluate effective scrubbing, gowning and gloving in the operating room setting (Table 1).^17^ A single operating room at a Level I trauma center was used for all data collection. The operating room adhered to national standards and guidelines (including the scrub sink outside of the room). Approved surgical attire were available, including surgical scrub brushes (Becton, Dickinson and Company, Franklin Lakes, New Jersey), surgical gowns (O&M Halyard, Inc., Alpharetta, Georgia), and surgical gloves (Cardinal Health, Dublin, Ohio). The individuals recruited consisted of medical students from the affiliated nationally renowned medical school with approximately 150 students per class – all of whom complete the surgical clerkships – and surgical residents, fellows and attendings from a wide variety of specialties. In the first phase of this research project, the modified Delphi technique was utilized to establish content validity and develop a checklist of 22 items for the process of surgical SGG.^17,18^

Participants were recruited and classified into three groups based upon prior surgical experience. Novices were defined as preclinical medical students with less than 8 weeks of surgical experience, intermediates were clinical medical students with at least 8 weeks of surgical experience and experts were residents or fellows with at least 6 months of postgraduate surgical training. Participants were recruited via email. A convenience sample of 20 participants per experience level was determined based on institution feasibility and similar previously reported studies.^11,19-21^ After obtaining informed consent, each study participant was assigned a unique study ID and completed a pre-test survey on demographics and prior surgical experience. The participant was then instructed to scrub, gown and glove in a staged inpatient operating room. The participants were not given any instruction or guidance on the task nor did they see the SGG checklist prior to performing the task. A scrub technician donned in surgical attire was available for the gowning and gloving portion of each trial. All necessary equipment was present at the scrub sink and with the scrub technician in the OR. Every participant was instructed to ask the scrub technician for each individual piece of equipment necessary to complete the task (towel, gown, gloves, etc.).

### Data Collection and Analysis

**Figure 1.**
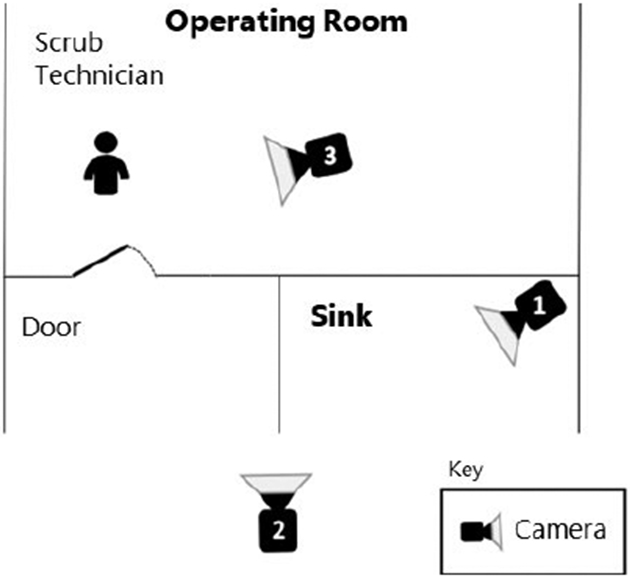
Schematic of Study Setup. Three cameras were placed to capture the entire procedure, including two outside the operating room at the scrub sink (Camera 1 and Camera 2) and one within the operating room (Camera 3). A scrub technician awaited inside the room for the gowning and gloving portion of the simulation.

Three cameras were placed to capture the entire procedure (Figure 1), including two outside the operating room at the scrub sink and one within the operating room. Participants were aware they were being video-recorded. The study investigators reviewed all recordings in order to render the videos de-identifiable by removing sound and facial features, while still capturing sufficient area above the neck to allow raters to assess if a mask was donned. Data collection occurred over a period of three months (February 2019 to May 2019).

Individual videos were scored according to the SGG checklist by two blinded raters with extensive surgical expertise. Both raters served as faculty in minimally invasive gynecologic surgery, with 6 and 9 years of surgical experience, respectively. Prior to rating the study videos, both surgeons were oriented to the study and SGG checklist by study personnel. The raters were provided with a written copy of the SGG checklist and a training video that described the correct steps and skills. Raters were blinded to subjects’ identity and prior surgical experience. Each rater watched the videos and graded the participants' scrubbing, gowning, and gloving performance according to the SGG checklist. The checklist is dichotomous, with steps appearing as “performed / not performed” (Table 1). If needed, the rater had the ability to stop, pause or rewind the video and watch again to ensure that the proper value was assigned to each step. All video scores and pre-study surveys were uploaded according to the assigned study ID to Research Electronic Data Capture (REDCap), a secure, web-based software platform for research studies (v 9.7.8).

For each participant, the completed SGG checklist items were summed to create an overall test score with a maximum value of 22. To assess inter-rater reliability of the overall test scores, we computed the intraclass correlation coefficient (ICC) from a mixed effects model with random effects for the subjects.^22^ ICC values range between 0 and 1, with less than 0.5 indicating poor reliability, between 0.5 and 0.75 indicating moderate reliability, values between 0.75 and 0.9 indicating good reliability, and values greater than 0.90 indicating excellent reliability.^23^ We also computed Cohen’s kappa (κ) to assess inter-rater reliability for each checklist item which should be interpreted as follows: values ≤ 0 indicate no agreement and 0.01-0.20 as none to slight, 0.21-0.40 as fair, 0.41-0.60 as moderate, 0.61-0.80 as substantial, and 0.81-1.00 as almost perfect agreement.^24, 25^

For the remaining analyses, we used the average of the reviewers’ scores for each participant. Cronbach’s alpha (α) was computed to determine the relatedness of the SGG checklist items or internal consistency of the test.^26^ The Cronbach’s a values for dichotomous checklists are interpreted as: α ≥ 0.7 as acceptable, 0.8 ≥ α ≥ 0.9 as good, and α ≥ 0.9 indicates high internal consistency.^27^ For each checklist item, we calculated the correlation between the individual item completion (averaged) and the test score (without the checklist item) to evaluate construct validity via Spearman rank correlation coefficient, which is a nonparametric measure of rank correlation. Correlations lower than 0.40, between 0.40 and 0.70, and greater than 0.70 were considered as weak, moderate and strong, respectively. The Wilcoxon rank sum test was used to determine discrimination validity of the overall test scores between all pairwise combinations of the novice, intermediate, and expert groups. Statistical analyses were performed using R software V3.6.0.

### Ethical Consideration

Formal approval for the study was obtained from the University of Pittsburgh School of Medicine’s Institutional Review Board (STUDY18100095). All students were invited to participate after providing informed consent. Confidentiality was maintained as no identifying information (only randomly assigned, non-consecutive Study ID numbers) was collected during the survey. The study code was kept on a password protected computer only accessible by the primary investigator.

## Results

### Demographics

We recruited 56 participants for this study including 18 novices, 19 intermediates and 19 experts (Table 2). 4 videos were excluded due to incidental incomplete captures during data collection (2 novice, 1 intermediate, and 1 expert). All of the novices reported scrubbing in ≤ 5 surgeries, 95% of intermediates reported scrubbing into 6-100 surgeries (5% scrubbed into ≥ 100), and all the experts reported scrubbing in ≥ 100 surgeries. Seventy percent of the experts reported confidence in the task, as opposed to only 11% of novices and intermediates.

### Reliability Outcome Measures (ICC, Cohen’s κ Spearman rank correlation coefficient)

**Figure 2.**
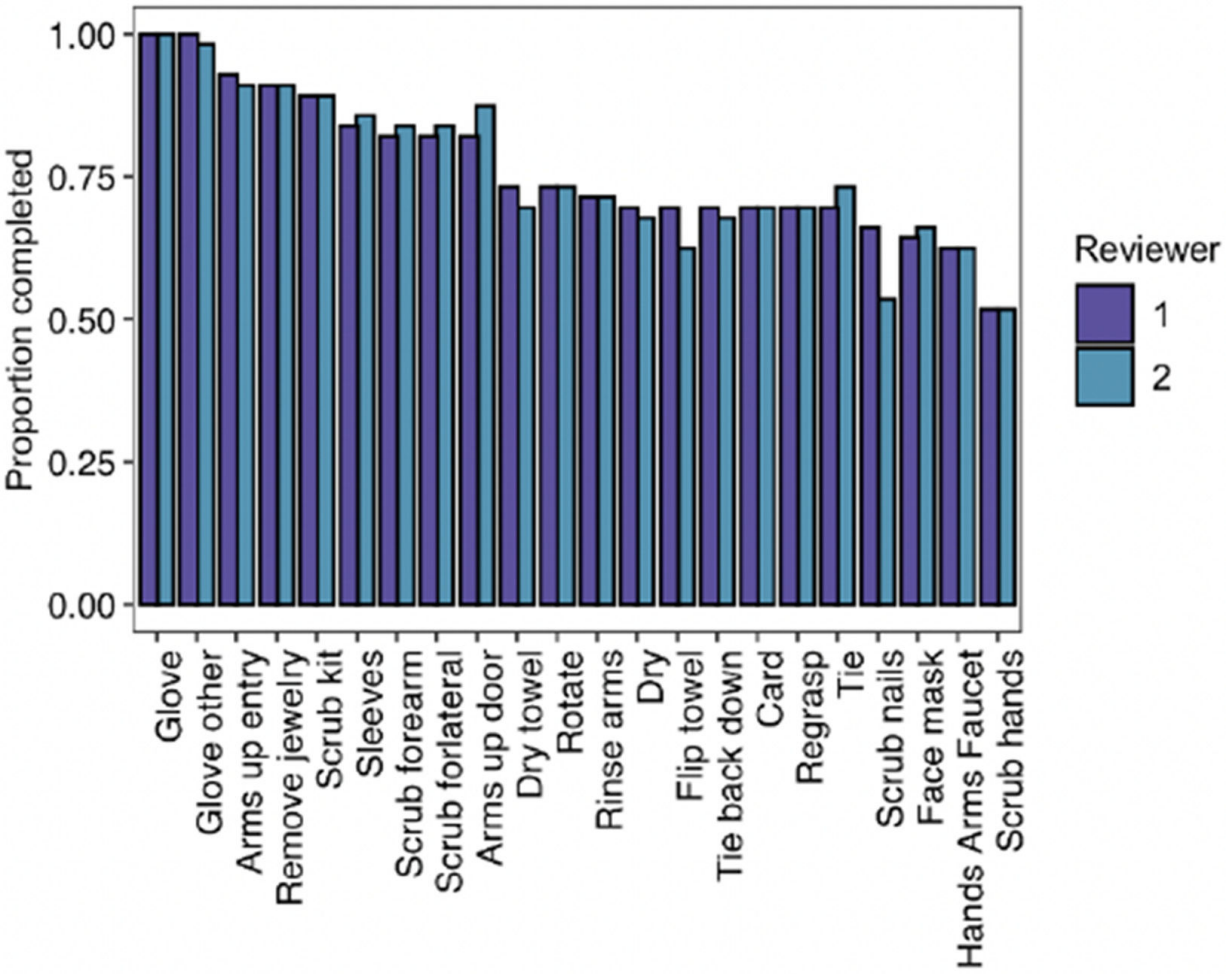
Proportion of Participants that Completed Checklist Items (as Evaluated by Reviewers).

**Figure 3.**
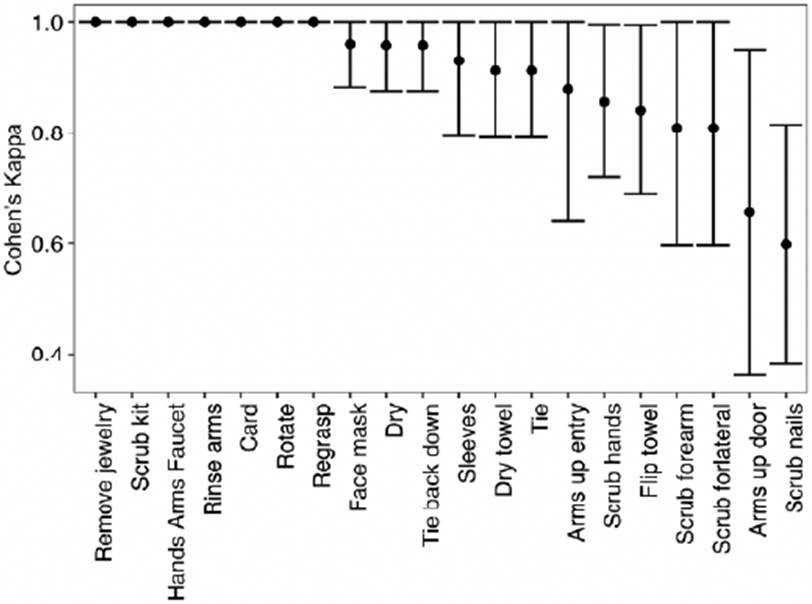
Cohen’s Kappa (κ) with 95% Confidence Intervals to assess Inter-rater Reliability for each Checklist Item. Values ≤ 0 indicate no agreement. Values 0.01-0.20 are interpreted as none to slight, 0.21-0.40 as fair, 0.41-0.60 as moderate, 0.61-0.80 as substantial, and 0.81-1.00 as almost perfect agreement.

**Figure 4.**
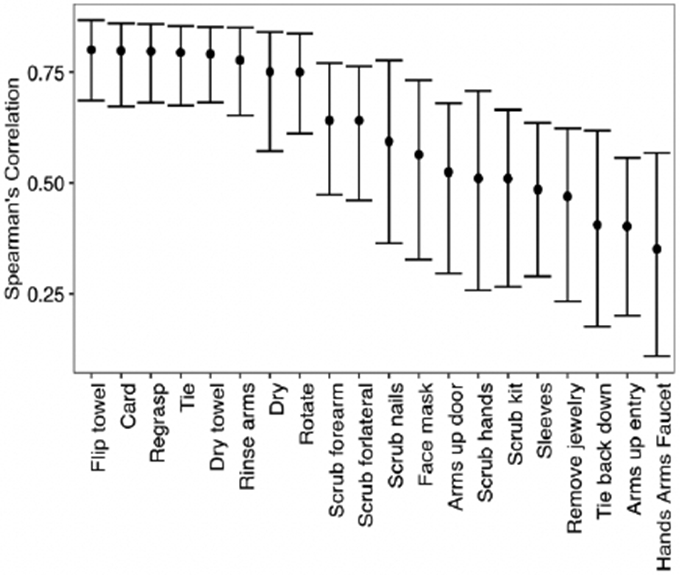
Spearman Rank Correlation Coefficient with 95% Confidence Intervals for each Checklist Item. The individual item completion (averaged) and the test score (without the checklist item) were correlated via the Spearman rank correlation coefficient to evaluate construct validity. Correlations lower than 0.40, between 0.40 and 0.70, and greater than 0.70 were considered as weak, moderate and strong, respectively.

The proportion of times the checklist item was marked completed by reviewers is demonstrated in Figure 2. The intraclass correlation coefficient was 0.990 (95% CI: [0.983, 0.994]) indicating a high level of agreement between reviewers. The inter-rater reliability for each item measured by Cohen’s κ ranged from 0.598 (scrubbing nails) to 1.00 (multiple measures) (Figure 3). Of note, two measures related to gloving were excluded, as they had no variation in completion. Further, the Spearman rank correlation coefficient of each checklist item and the overall score ranged from 0.351 to 0.801, with the gloving measures also excluded from this analysis (Figure 4). Of the remaining 20 checklist items, 11 demonstrated moderate correlation and 8 demonstrated strong correlation. This indicates that the checklist has a moderate to high level of construct validity.

### Validity Outcome Measures (Cronbach α and Wilcoxon rank sum test)

**Figure 5.**
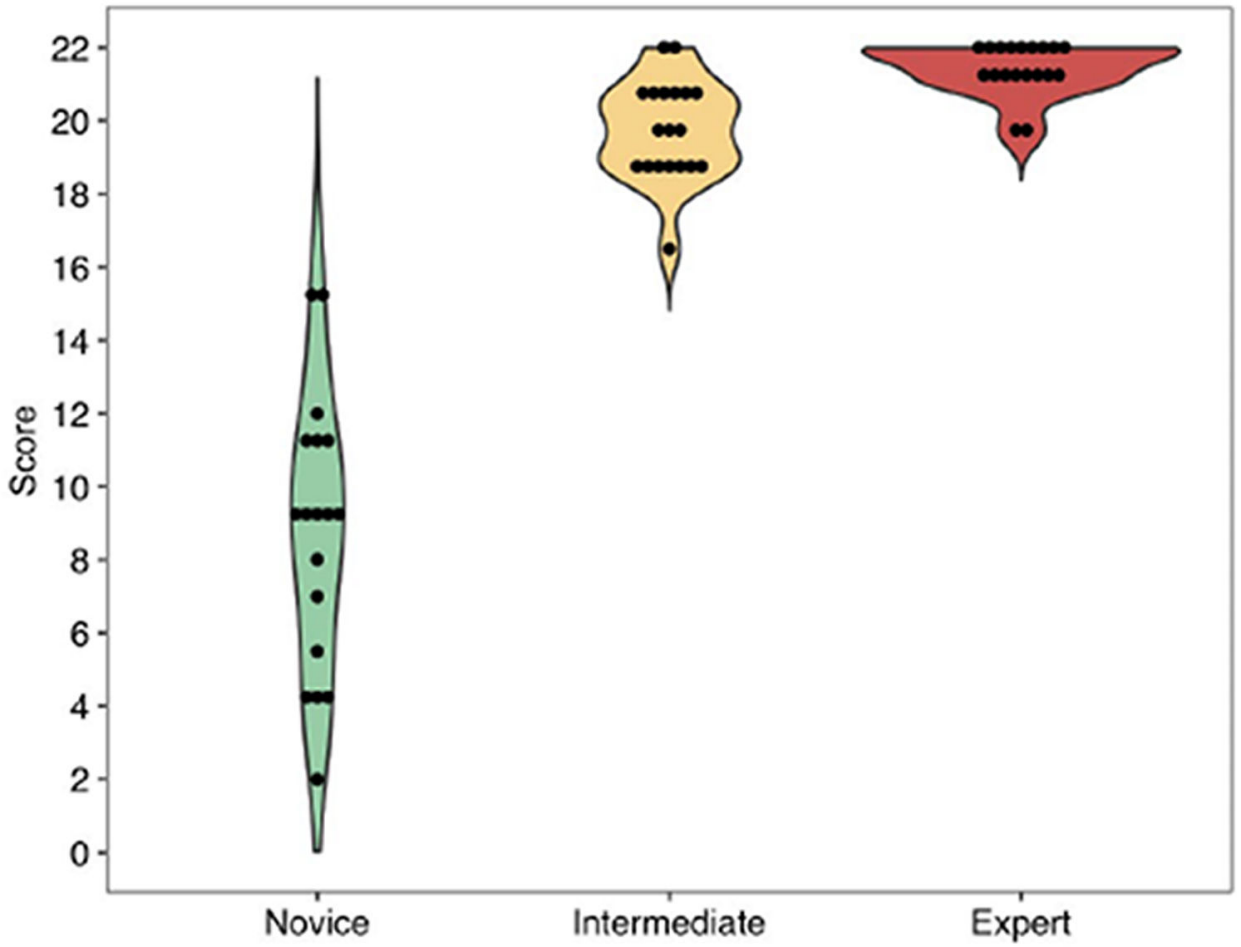
Distribution of Overall Test Scores by Expertise Level. The median test score was 9 among novices, 20 among intermediates, and 21.5 among experts. There was greater variability in scores among the novices than the intermediates and experts. All groups differed significantly in the distributions of their test scores (pairwise p-values all <0.001).

The internal consistency of the test measured by Cronbach’s α was 0.950 (95% CI [0.944, 0.952]), indicating a high level of correlation among test items. The overall median test score was 19.7 with an interquartile range of 11.4-21.1. The median test score was 9 among novices, 20 among intermediates, and 21.5 among experts (Figure 5). There was greater variability in scores among the novices than the intermediates and experts. All groups differed significantly in the distributions of their test scores.

## Discussion

We found that our 22-item, task-based SGG checklist demonstrates good reliability and discriminant validity. This checklist has a high inter-rater reliability and good internal consistency. Inter-rater reliability measures the level of agreement between independent observers. It reveals unambiguity of the checklist and the optimization of its practical use by minimizing the effect of the observer variability. The SGG checklist also demonstrates discriminant validity by detecting a difference in skills between learners with different levels of surgical experience. Good discriminant validity, a subtype of construct validity, ascertains whether two supposedly unrelated constructs are actually unrelated.

The ICC (0.99) indicates excellent overall inter-rater reliability of the checklist. The item inter-rater reliability was > 0.6 for all items, with 82% of the items > 0.8, indicating that there was substantial to near perfect agreement for many of the checklist items. Item discrimination is typically low for easy and difficult checklist items because all participants perform similarly on them. Two of the items (right and left glove) were excluded for this reason; there was no variation because every participant completed the item. The SGG checklist demonstrates discriminant validity by detecting a difference in skill between all three groups, particularly for novices compared to intermediates and experts (Figure 5). This result provides some support for construct validity, which is an important step in the initial evaluation of an assessment tool and internal validity. Further, the Cronbach α was above the traditional cutoff of 0.7,^27,28^ suggesting excellent internal consistency.

To our knowledge, this is the first study to assess the reliability and discriminant validity of a developed, consensus-based checklist for the skill of scrubbing, gowning and gloving. Current methods of teaching include formal instruction prior to clinical rotations, detailed written protocols and videos of the process.^2, 3^ Other resources are available online, such as guidelines from the Association of PeriOperative Registered Nurses, however the references are only accessible via paid membership.^29^ Pirie et al. provides a 6-step hand washing and gowning and gloving method, but the discrete steps for gowning and gloving are not provided.^2,3^ Additionally, the methods mentioned only serve to inform students; there are no resources available that provide preparation or standardized assessment of students’ understanding of the procedures.^30^

Our results show that novices have a significantly lower baseline skillset (median score of 9) compared to intermediates and experts (median score of 20 and 21.5, respectively). This suggests that the implementation of this SGG checklist would be effective for both learning and assessment. Medical students could benefit from a simulation model informed by the SGG checklist at the start of their clerkship rotations. There is evidence that providing simulation education prior to OR experiences give students increased confidence and comfort,^15,31-33^ which can mitigate stress that hinder learning.^4-6^ As an assessment tool, the SGG checklist can be used within curriculums after surgical clerkships via objective structured clinical examinations (OSCEs). Post-clerkship, students would be expected to perform at an expert level to pass.

While our checklist demonstrates good reliability and validity, it is important to recognize the tradeoffs between checklists and global rating scales (GRSs) in medical education. The advantages and disadvantages of each have long been debated.^13,34-37^ In general, checklists assess *whether or not the task was done* (washed hands), whereas rating scales assess *how well tasks were performed* (washed hand in fluent, efficient manner).^35^ Checklists are advantageous for their ease-of-use and the step-by-step nature makes them particularly useful for raters that are less familiar with the evaluated skill.^38^ Although checklists seem to be a more objective measure, there is some evidence that the dichotomous nature of checklists may result in a loss of information, and may prioritize thoroughness over clinical competence.^34,39-43^ GRSs are more sensitive for detecting differing levels of experience and allow raters to have more flexibility on the assessment of more complex, diverse tasks.^44-47^ An accurate global assessment requires rater judgements and decision-making, rendering it dependent upon rater characteristics (clinical expertise and familiarity) and task complexity.^48-50^ This may be disadvantageous in a high-stakes assessment setting.^48,49^ In a systematic review comparing global rating scales versus checklists in simulation-based assessments, interrater reliability was high (similar to our study) and slightly better for checklists, without differences in discrimination and correlation with other measures.^13^ They also reported that GRS are useful for assessment across multiple tasks (such as an OSCE), with high average inter-item and inter-station reliability.^13^ A checklist is ideal for evaluation of SGG because it is a single task that does not require a high level of rater expertise.

Our study has many strengths. The SGG checklist was developed using the Delphi technique in our prior study,^17^ a widely accepted technique in medical education and quality improvement.^51-53^ The reviewers were blinded and were provided de-identified videos to minimize bias. An actual, functioning OR setting was used to increase the strength of study, specifically external validity. The expertise groups were well-distributed, and the survey characteristics also correlated well with surgical expertise. While the term *validity* must be used cautiously in the realm of medical education, ^44,54-55^ our results show that the SGG checklist is able to discriminate between learners of novice, intermediate, and expert level.

Limitations of our study include the single-center design which decreases external validity. Use of a convenience sample can potentially introduce a selection bias if factors leading to participation affected the checklist performance. However, study participants were stratified based on experience alone and the study should be minimally affected by this sampling method. Also, the study has potential inherent Hawthorne bias given that they participants were aware that they were being evaluated and recorded. Our checklist does not take into account the weight of particular items because failure of any one of the items on the SGG checklist should equate to overall failure in the pre-operative setting. This is particularly important for scrubbing, gowning and gloving because failure warrants immediate restart of the process (i.e., re-scrub, gown and glove).

We describe the development of a reliable and valid SGG checklist intended to enhance medical education curricula, specifically to inform a simulated scrubbing, gowning and gloving activity. There is also evidence that this can be used as an assessment tool within an OSCE or other standardized medical education exams. Future steps include further validation (criterion, convergent and predictive) of the SGG checklist, multi-center testing, and implementation into a medical education curriculum.

## Figures and Tables

**Table 1.** Scrubbing, Gowning and Gloving (SGG) Checklist.

Scrubbing
Remove all jewelry Put on face mask Grab a pre-package scrub/nail kit Moisten hands and arms under the water without touching the faucet Use firm/bristled side of brush to scrub nails Use firm/bristled end of scrub brush to scrub all surfaces of fingers Use sponge to scrub the entire length of forearm, starting most distal (wrist) to elbow Use sponge to scrub entire length of contralateral forearm, starting most distal (wrist) to elbow Rinse off both arms Use back/butt/hip to enter OR Gowning and Gloving Enter OR with elevated hands/arms taking care to avoid touching anything Hold out one hand to accept a dry towel from scrub tech/nurse Dry opposite hand/arm using the hand the towel was placed in Dry opposite hand/arm that has not yet been dried With scrub tech/nurse holding gown open, place both hands/arms into sleeves Allow nonsterile nurse/circulator to tie up back of gown With scrub tech/nurse holding right glove open, put hand into right glove With scrub tech/nurse holding left glove open, put left hand into glove Hand card to scrub tech/nurse or circulator Rotate in gown with scrub tech/nurse or circulator still holding card Regrasp the tie from the scrub tech/nurse or circulator Tie both ties of gown together

**Table 2.** Baseline Demographic Variables.

Variable	Overall (n = 56)	Novice (n = 18)	Intermediate (n = 19)	Expert (n = 19)
Age median	27	25	27	29
Male, n (%)	23 (41%)	8 (44%)	12 (63%)	3 (16%)
Number of surgeries, n (%)				
0-5	18 (32%)	18 (100%)	0 (0%)	0 (0%)
6-25	3 (5%)	0 (0%)	3 (16%)	0 (0%)
26-50	6 (11%)	0 (0%)	6 (32%)	0 (0%)
51-100	9 (16%)	0 (0%)	9 (47%)	0 (0%)
101+	21 (37%)	0 (0%)	1 (5%)	20 (100%)
I feel confident about my ability to scrub, n (%)				
Disagree or Strongly Disagree	21 (37%)	15 (83%)	4 (21%)	2 (10%)
Neutral	18 (32%)	1 (6%)	13 (68%)	4 (20%)
Agree or Strongly Agree	18 (32%)	2 (11%)	2 (11%)	14 (70%)
I think the operating room is a comfortable learning environment n (%)				
Disagree or Strongly Disagree	21 (37%)	9 (50%)	5 (26%)	2 (10%)
Neutral	18 (32%)	6 (33%)	10 (53%)	8 (40%)
Agree or Strongly Agree	18 (32%)	3 (17%)	4 (21%)	10 (50%)
Has surgical career interest, n (%)				
I don’t know	5 (9%)	5 (28%)	0 (0%)	0 (0%)
No	14 (25%)	4 (22%)	10 (53%)	0 (0%)
Yes	38 (67%)	9 (50%)	9 (47%)	20 (100%)
